# Nrf2/HO-1-sulfiredoxin1 pathway involved in nanobubble hydrogen-dissolved water-mediated protective effects by ultrasound-guided early local injection in a rat model of chronic constriction injury-induced neuropathic pain

**DOI:** 10.3389/fnmol.2025.1666575

**Published:** 2025-12-17

**Authors:** Junmin Yu, Zixiao Yin, Hongmin Ma, Bing Zhang, Chao Meng

**Affiliations:** 1 Department of Pain, Affiliated Hospital of Qingdao University, Qingdao, China; 2 Department of Radiotherapy Center, Affiliated Hospital of Qingdao University, Qingdao, China; 3 Department of Pain, The Third People’s Hospital of Qingdao, Qingdao, China; 4 Department of Anesthesiology, Affiliated Hospital of Qingdao University, Qingdao, China

**Keywords:** nanobubble hydrogen-dissolved water, chronic constriction injury, neuropathic pain, ultrasound-guided, Nrf2/HO-1, sulfiredoxin1

## Abstract

**Introduction:**

Neuropathic pain (NP) is a kind of common and intractable chronic pain. Hydrogen (H_2_)-rich water exhibited protective effects in NP by intrathecal injection, drinking, and intraperitoneal injection. The nanobubble H2-dissolved water (NHW) is a solution that contains H_2_ bubbles and H_2_ in lysis state. Therefore, this study aimed to observe the effects of ultrasound-guided local injection with NHW in the model of NP, and try to find its possible mechanism.

**Methods:**

The rat sciatic nerve was ligated to establish chronic constriction injury (CCI)-induced NP model. The CCI rats received NHW at low or high concentrations 1 or 3 times (*n* = 6). During the experiment, the paw withdrawal thresholds (PWT) and paw withdrawal latency (PWL) were detected. At 14 days after CCI, the organizational structure of nerve, inflammatory response, and oxidative stress damage were measured. Additionally, the Nrf2/HO-1 and sulfiredoxin-1 were also detected by western blotting and RT-PCR.

**Results:**

Compared with low concentration, in the high concentration group, the PWT and PWL were attenuated on Day 1, 3, 5, 7, and 14 after CCI (*p* < 0.05). On Day 14, nerve injury, inflammatory response, and oxidative stress injury were relieved significantly in high concentration than at low concentration, and the effect was greater at multiple doses (3 times) at high concentrations (*p* < 0.05), as were the increase in the protein and mRNA levels of Nrf2/HO-1 and sulfiredoxin-1.

**Conclusion:**

Ultrasound-guided early local injection of NHW attenuated sciatic nerve injury, alleviated mechanical allodynia and thermal hyperalgesia and inhibited inflammation and oxidative stress damage via the Nrf2/HO-1-sulfiredoxin1 pathway in a rat model of CCI.

## 1 Introduction

In 2008, neuropathic pain (NP) was defined as “pain caused by a lesion or disease in the peripheral or central nervous system,” which is characterized by abnormal sensations, including dysesthesia, hyperalgesia, and allodynia (Colloca et al., 2017; Bouhassira et al., 2008). NP is a common and intractable form of chronic pain, and it has a severely negative effect on quality of life, including not only physical health but also mental health, which places a heavy burden on society and families (Petzke et al., 2022; Bader et al., 2022). The prevalence of NP is 5–10%, and millions of people suffer from new-onset NP every year (Martínez-Serrat et al., 2022). Thus, a treatment for NP is urgently needed.

Currently, a number of therapeutic strategies, including drugs, exercise, psychotherapy, and interventional therapy, are used in the clinic to treat NP, but only 30% of patients achieve satisfactory pain relief (Sumizono et al., 2022; Zhang et al., 2022). Although pharmacotherapy is the basis of NP treatment, the side effects cause many patients to discontinue treatment (Finnerup et al., 2015). Therefore, it is necessary to explore alternatives for the treatment of NP.

Hydrogen (H_2_), a small molecule colorless and odorless gas, was previously thought to be a biologically inert gas (Li et al., 2018). In 2007, H_2_ was shown to have an anti-oxidative effect against brain ischemia–reperfusion injury, which attracted intense interest from researchers (Ohsawa et al., 2007). The protective effects of H_2_, such as anti-inflammatory, anti-apoptotic, and anti-oxidative damage effects, have been reported in many diseases, such as lung diseases, skin diseases, heart diseases, and brain diseases (Ishibashi, 2019). H_2_ treatment was even included in the Chinese treatment of COVID-19 (Luo et al., 2022). There are three main forms of H_2_: gas, H_2_-rich water, and nanomaterials. Several previous studies have applied H_2_ in the treatment of NP. In the early stage of research, intrathecal injection of H_2_-rich water decreased oxidative stress in a rat NP model (Chen et al., 2013), then the researchers found that drinking H_2_-rich water also attenuated NP in mice (Kawaguchi et al., 2014; Lian et al., 2021). However, in the later stage of research, H_2_-rich water administered by intraperitoneal injection was often used in the treatment of NP (Wang et al., 2018; Coral-Pérez et al., 2022; Chen et al., 2019). However, it has certain disadvantages, such as the volatility of H_2_ and the strict storage conditions. Moreover, these methods are difficult for patients with NP to accept in the clinical. Therefore, we proposed a method of local injection around the nerve, which was closer to standard clinical practice.

Compared with H_2_-rich water, nanobubble H_2_-dissolved water (NHW) has multiple advantages. NHW is a solution that contains H_2_ bubbles (less than 1 μm in diameter) (Kato et al., 2015), and not only the nanobubble state of H_2_ but also the lysis state of H_2_ in the solution greatly increase the H_2_ concentration (Tanaka et al., 2022). Additionally, NHW is more stable (Tanaka et al., 2022). Therefore, the aim of this study was to investigate the effects of ultrasound-guided local injection of NHW to the sciatic nerve in a rat model of chronic constriction injury (CCI)-induced NP and to elucidate the underlying mechanism.

## 2 Methods and materials

### 2.1 Animals

The Sprague–Dawley (SD) rats (male, 8 weeks old, 200 g) used in this study were acquired from the Animal Center of the Affiliated Hospital of Qingdao University. All the rats had free access to food and water and were housed at room temperature (24 ± 1 °C) under a 12 h light/dark cycle in separate cages. All animal care and experimental procedures were approved by the Animal Care and Welfare Committee of the Affiliated Hospital of Qingdao University and complied with the ARRIVE guidelines (AHQU-MAL20241224MC).

### 2.2 NHW production

NHW was produced using an NHW-producing apparatus (NB-T71A; Shanghai Nanobubble Technology Co. Ltd., Shanghai, China). The apparatus produced H_2_ through electrolysis of ultrapure water, separated H_2_ nanobubbles, and finally made NHW; the H_2_ concentration could reach a maximum of 2,600 ppb in the NHW. The NHW was stored in an aluminum bag at room temperature and sterilized by *γ* radiation.

### 2.3 CCI model

A rat CCI model was established to induce NP as described in a previous study (Bennett and Xie, 1988). After the rats were anesthetized by intraperitoneal injection of sodium pentobarbital (50 mg/kg), the left sciatic nerve was exposed between the biceps femoris and gluteus superficialis. Then, the left sciatic nerve was loosely ligated using 4/0 silk with 4 ligature threads at 1 mm intervals. The entire procedure was completed in a sterile environment, and the rats were given topical antibiotics after surgery. The rat CCI model was established on Day 0.

### 2.4 Groups

**Figure 1:**
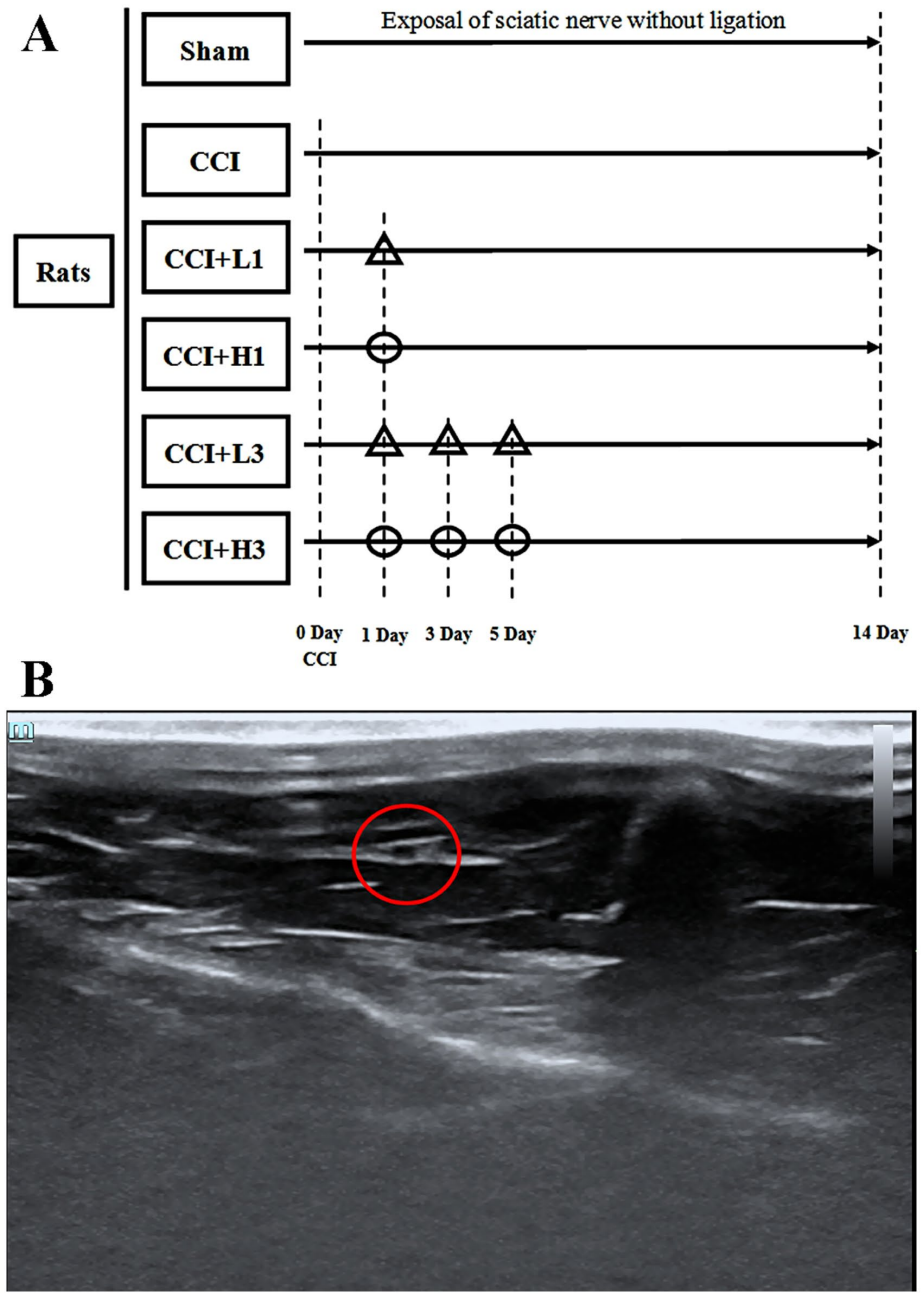
**(A)** Study design. CCI: chronic constriction injury; *Δ*: low-concentration nanobubble hydrogen-dissolved water (1,300 ppb) injected into the sciatic nerve under ultrasound; *Ο*: high-concentration nanobubble hydrogen-dissolved water (2,600 ppb) injected into the sciatic nerve under ultrasound. **(B)** Sciatic nerve under ultrasound. The sciatic nerve is represented by a red circle.

After the CCI model was established, 36 rats were randomly divided into the following groups (*n* = 6 per group); (1) sham group: rats who had the were left sciatic nerve exposed but not ligated; (2) CCI group: rats with the established CCI model; (3) CCI + low concentration NHW once group (CCI + L1 group): rats with the established CCI model who were injected locally with low concentration NHW (1,300 ppb, 5 mL/kg) under ultrasound on Day 1 after CCI; (4) CCI + high concentration NHW once group (CCI + H1 group): rats with the established CCI model who were injected locally with high concentration NHW (2,600 ppb, 5 mL/kg) under ultrasound on Day 1 after CCI; (5) CCI + low concentration NHW with three times group (CCI + L3 group): rats with the established CCI model who were injected locally with low concentration NHW (1,300 ppb, 5 mL/kg) under ultrasound on Day 1, 3, 5 after CCI; (6) CCI + high concentration NHW three times group (CCI + H3 group): rats with the established CCI model who were injected locally with high concentration NHW (2,600 ppb, 5 mL/kg) under ultrasound on Day 1, 3, 5 after CCI (Figure 1A).

### 2.5 Ultrasound-guided local injection into the rat sciatic nerve

We performed local injection via ultrasound according to the method described in a previous study (Wu et al., 2023). Ultrasound imaging (Shenzhen Mindray Biomedical Electronics Co., Ltd., Shenzhen, China) was performed with a high-frequency probe (bandwidths of 15–30 MHz) on the abdominal side of the left hind limb of the rats under 2% isofurane anesthesia (Figure 1B). The fur in the observation area was cleaned and disinfected locally with iodine before puncture.

### 2.6 Behavioral tests

The behavioral tests were performed at the baseline (1 day before CCI) and on Day 0, 1, 3, 5, 7, and 14 after CCI. A Von-Frey pain test kit (CA91367; San Diego Scientific Instruments, Inc., CA, USA) was used to measure the paw withdrawal threshold (PWT), which was considered mechanical allodynia. A hot plate (Shanghai Xinruan Information Technology Co., Ltd., Shanghai, China) was used to measure the paw withdrawal latency (PWL), which was considered thermal hyperalgesia. A rotarod system (IITC Life Science Inc., CA, USA) was used to measure motor function.

The PWT was determined as follows: The rats were placed in a cage with metal mesh. The rats were allowed to adapt to the cage for 15 min until they were quiet. A Von-Frey wire was used to stimulate the middle of the rats’ left hind paw vertically with sufficient force to bend the wire into an S shape for 5 s. When the rats withdrew their hind paw, the force corresponding to the thickness of the Von-Frey wire was recorded. The maximum force was that of a 26 g Von-Frey wire. This test was performed 3 consecutive times with an interval of 5 min, and the average PWT was recorded (Wang et al., 2018; Coral-Pérez et al., 2022).

The PWL was determined as follows: the rats were placed on a hot plate and allowed to adapt for 15 min until they were quiet. The temperature of the hot plate was then set to 55 ± 0.1 °C. When the rat started licking its left hind paw, the time was recorded. The maximum time allowed was 25 s. This test was performed 3 consecutive times with an interval of 10 min, and the average PWL was recorded (Wang et al., 2018; Coral-Pérez et al., 2022).

Motor function was determined as follows: The rats were placed on the rotarod system. The system was started at a speed of 5 rpm and accelerated constantly to 20 rpm. When the rat fell off, the time was recorded. The maximum time was 300 s. This test was performed 3 consecutive times with an interval of 15 min, and the average motor function was recorded (Wang et al., 2018; Coral-Pérez et al., 2022).

### 2.7 Histopathological staining

On Day 14 after CCI, the rats were euthanized. The sciatic nerve at the site of ligation in each group was harvested and fixed with formaldehyde for 72 h. After alcohol dehydration, the tissues were embedded in paraffin and cut into 5 μm sections. The sections were stained with hematoxylin and eosin and observed under an optical microscope (Olympus Corporation, Tokyo, Japan).

### 2.8 Enzyme-linked immunosorbent assay (ELISA)

On Day 14 after CCI, the rats were euthanized. The sciatic nerve at the site of ligation and the L5 dorsal root ganglion (DRG) in each group were harvested and stored at −80 °C. The tissues were homogenized in 4 °C lysis buffer by sonication and incubated on ice for 30 min. Then, the lysate was clarified by centrifugation at 13,000 g at 4 °C for 10 min. The supernatants were collected for detection of the interlenkin (IL)-1β, IL-6, IL-10, and tumor necrosis factor (TNF)-*α* concentration following the instructions of the respective ELISA kits (Thermo Fisher Scientific Inc., Massachusetts, USA).

### 2.9 Oxidative stress injury

The supernatants of the sciatic nerve and DRG were also used to assess oxidative stress injury by detecting the content of superoxide dismutase (SOD), malondialdehyde (MDA), catalase (CAT), and 8-hydroxydeoxyguanosine (8-OHDG) according to the instructions of the relative kits (Nanjing Jiancheng Bioengineering Research Institute Co. Ltd., Nanjing, China).

### 2.10 Western blotting

On Day 14 after CCI, the rats were euthanized. The L5 DRGs in each group were harvested and stored at −80 °C. The tissues were crushed under liquid nitrogen and dissolved in lysis buffer (containing 1 mM phenylmethanesulfonylfluoride) for 10 min. Then, the mixture was centrifuged at 14,000 g at 4 °C for 10 min, and the supernatants were boiled for 10 min to obtain the samples. First, the protein concentration was determined by the bicinchonininc acid (BCA) method (Nanjing Jiancheng Bioengineering Research Institute Co. Ltd.), after which the proteins were separated by the sodium dodecyl sulfate-polyacrylamide gel electrophoresis (SDS-PAGE). After the proteins were transferred to a polyvinylidene fluoride (PVDF) membrane, the membrane was blocked with 5% skim milk. Next, the membrane was incubated with primary antibodies (Thermo Fisher Scientific Inc.) against nuclear factor erythroid 2-related factor 2 (Nrf2, 1:1500), heme oxygenase-1 (HO-1, 1:1000), and sulfiredoxin-1 (1:500) overnight at 4 °C. Then, the membrane was incubated with the secondary antibody (1:4000, Beyotime Biotech Inc., Shanghai, China) for 1 h at room temperature. Finally, the membrane was developed via enhanced chemiluminescence (Beyotime Biotech Inc.) using Quantity One software (Bio-Rad Laboratories, Inc., Shanghai, China). *β*-actin was used as a control (Coral-Pérez et al., 2022).

### 2.11 Real-time RT-PCR

Total mRNA was extracted from DRG samples using TRIzol reagent (Invitrogen, Carlsbad, CA, USA) according to the manufacturer’s instructions. cDNA synthesis was performed using PrimeScript RT Master Mix (Takara, China), and RT-PCR was performed using SYBR Green Supermix (Bio-Rad, Hercules, CA, USA). The primer sequences were as follows: Nrf2 forwards and reverse primers were 5’-TGAAGCTCAGCTCGCATTGA-3′ and 5’-TGCTCCAGCTCGACAATGTT-3′; HO-1 forwards and reverse primers were 5’-ATCGTGCTCGCATGAACACT-3′ and 5’-CCAACACTGCATTTACATGGC-3′; and sulfiredoxin-1 forwards and reverse primers were 5’-GTGCACAACGTACCAATCG-3′ and 5’-GCCCCCAAAGGAATAGTAGTAG-3′. The data are expressed as relative Ct values to β-actin (forwards: 5’-CTGAATGGCCCAGGTCTGAG-3′; reverse: 5’-AAGTCAGTGTACAGGCCAGC-3′) and the results are expressed as 2^-ΔΔCt^.

### 2.12 Statistical analysis

All the data are expressed as the mean ± standard deviation (SD). Differences between multiple groups were analyzed by two-way analysis of variance (ANOVA) with a *post hoc* Tukey test for PWT, PWL, and motor function. Differences between multiple groups were analyzed by one-way ANOVA and Bonferroni test was used for post comparisons for WB, inflammation and oxidative stress injury. *p* < 0.05 was considered to indicate a significant difference. SPSS 17.0 software (SPSS, Chicago, Illinois, USA) was used for the analyses.

## 3 Results

### 3.1 Characteristics

In this study, 36 rats were used. With the exception for 6 rats in which the sciatic nerve was exposed without ligation (the controls), the CCI model was successfully established in the remaining 30 rats. Therefore, the data from all 36 rats were included in the statistical analysis.

### 3.2 NHW attenuated CCI-induced mechanical allodynia and thermal hyperalgesia

**Figure 2:**
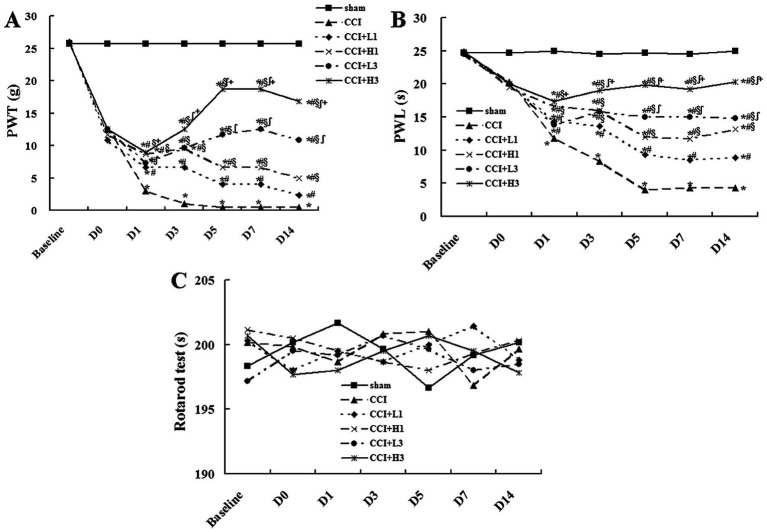
The behavioral tests results (*n* = 6; means). **(A)** paw withdrawal threshold (PWT); **(B)** paw withdrawal latency (PWL); **(C)** motor function determined by the rotarod test. To avoid confusion in the figures, the standard deviation is not displayed to clarify the figures. ^*^p < 0.05 vs. the sham group; ^#^*p* < 0.05 vs. the CCI group; ^§^*p* < 0.05 vs. the CCI + L1 group; ^∫^*p* < 0.05 vs. the CCI + H1 group; ^+^*p* < 0.05 vs. the CCI + H1 group.

The data were analyzed by two-way ANOVA first, and then analyzed with Tukey test for differences between groups. At baseline, the PWT and PWL in all groups were similar. Compared with those in the sham group, the PWT and PWL were significantly lower on Day 0, 1, 3, 5, 7, and 14 after CCI. However, in the CCI group, the PWT and PWL on Day 1, 3, and 5 after CCI continued to decrease and remained stable to Day 7 and 14. On Day 0, the PWT and PWL did not significantly differ among the CCI, CCI + L1, CCI + H1, CCI + L3, and CCI + H3 groups. On Day 1 after CCI, the PWT and PWL in the CCI + L1, CCI + H1, CCI + L3, and CCI + H3 groups were greater than those in the CCI group (*p* < 0.05), and those in the CCI + H1 and CCI + H3 groups were greater than those in the CCI + L1 and CCI + L3 groups (*p* < 0.05). The PWT and PWL were not significantly different between the CCI + H1 and CCI + H3 groups or between the CCI + L1 and CCI + L3 groups. On Day 3 after CCI, the PWT and PWL in the CCI + L3 and CCI + H3 groups were greater than those in the CCI + L1 and CCI + H1 groups (*p* < 0.05), respectively, and those in the CCI + H3 group were greater than those in the CCI + L3 group (*p* < 0.05). The PWT and PWL in the CCI + H1 group were greater than those in the CCI + L1 group (*p* < 0.05). The same trend was also found On Day 5, 7 and 14 after CCI (Figure 2). Furthermore, motor function was not affected (as determined by the rotarod test) in any of the groups (Figure 2).

### 3.3 NHW alleviated the CCI-induced nerve injury

**Figure 3:**
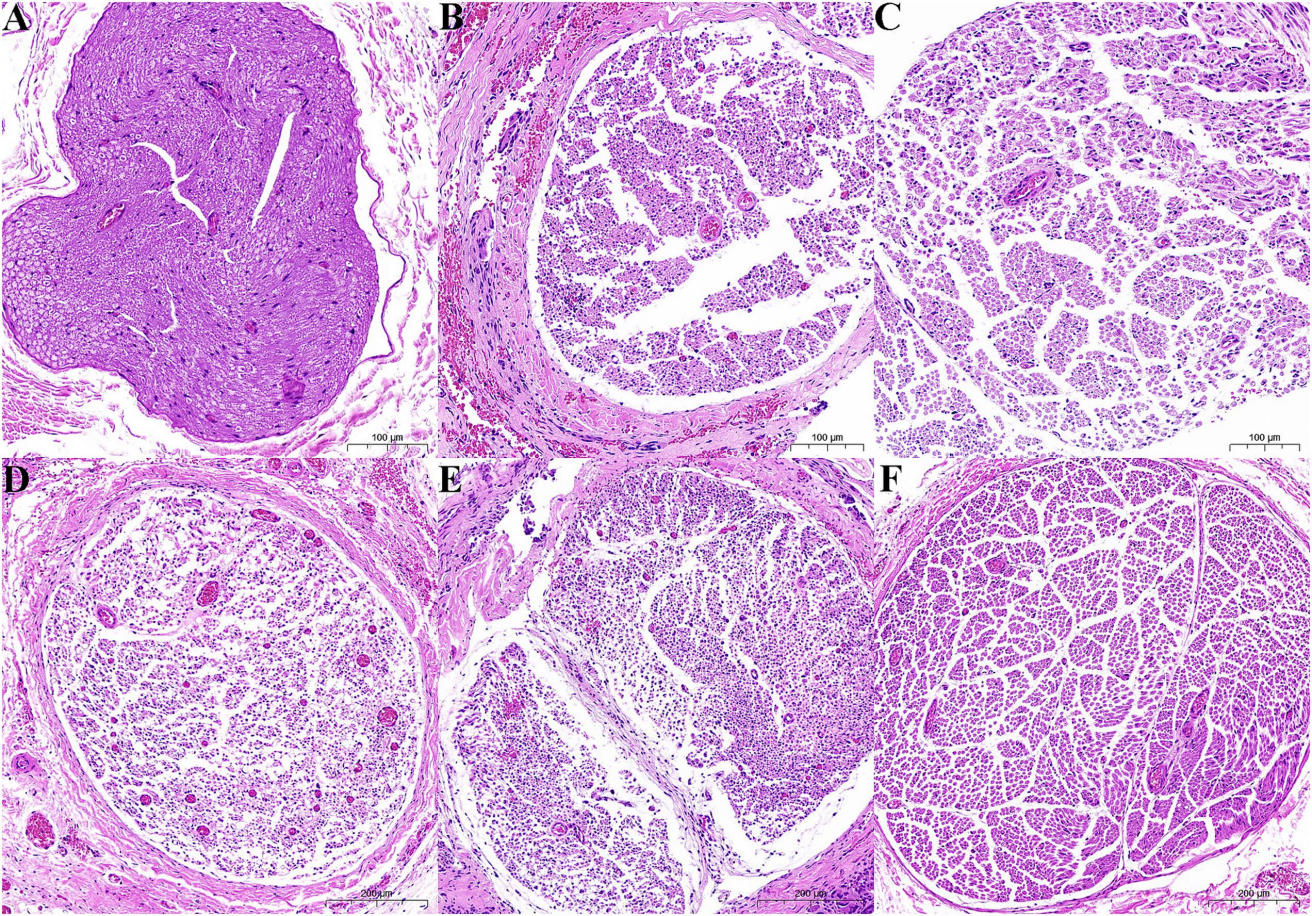
Tissue morphology of the rat sciatic nerve (HE staining, ×10). **(A)** Sham group; **(B)** CCI group; **(C)** CCI + L1 group; **(D)** CCI + H1 group; **(E)** CCI + L3 group; **(F)** CCI + H3 group. The axon distribution was uniform and full in the sham group, whereas the number of axons was sparse with varying sizes, thicknesses, and shapes in the CCI group. After the nanobubble hydrogen-dissolved water was injected, the number of axons increased, and the distribution tended to be neat, which was most evident in the CCI + H3 group.

In the sham group, the sciatic nerve exhibited a well-organized structure as demonstrated by HE staining. The nerve fibers were arranged in a parallel and orderly manner. The myelin sheaths surrounding the axons were intact and displayed a clear and uniform appearance. HE staining revealed that the sciatic nerve was significantly altered in the CCI group. The nerve fibers appeared disorganized and disrupted. There were areas of myelin sheath breakdown and degeneration of axons. However, injury to the sciatic nerve was alleviated in the CCI + L1, CCI + H1, CCI + L3, and CCI + H3 groups, and injury to the sciatic nerve was lightest in the CCI + H3 group (Figure 3).

### 3.4 NHW decreased CCI-induced nerve inflammation

**Figure 4:**
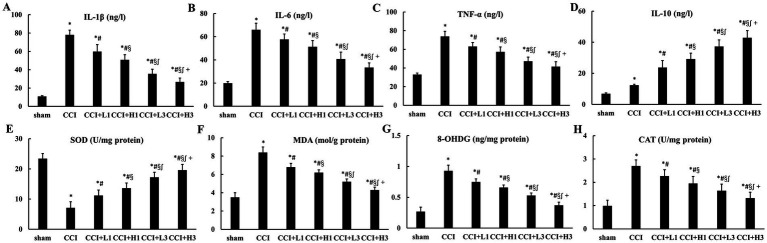
The inflammation and oxidative stress injury in nerve (*n* = 6, means ± SD). **(A)** IL-1β; **(B)** IL-6; **(C)** TNF-α; **(D)** IL-10; **(E)** SOD; **(F)** MDA; **(G)** 8-OHDG; **(H)** CAT. CCI: chronic constriction injury; IL: interlenkin; TNF: tumor necrosis factor; SOD: superoxide dismutase; MDA: malondialdehyde; 8-OHDG: 8-hydroxydeoxyguanosine; CAT: catalase. ^*^p < 0.05 vs. sham group; ^#^*p* < 0.05 vs. CCI group; ^§^*p* < 0.05 vs. CCI + L1 group; ^∫^*p* < 0.05 vs. CCI + H1 group; ^+^*p* < 0.05 vs. CCI + L3 group.

**Figure 5:**
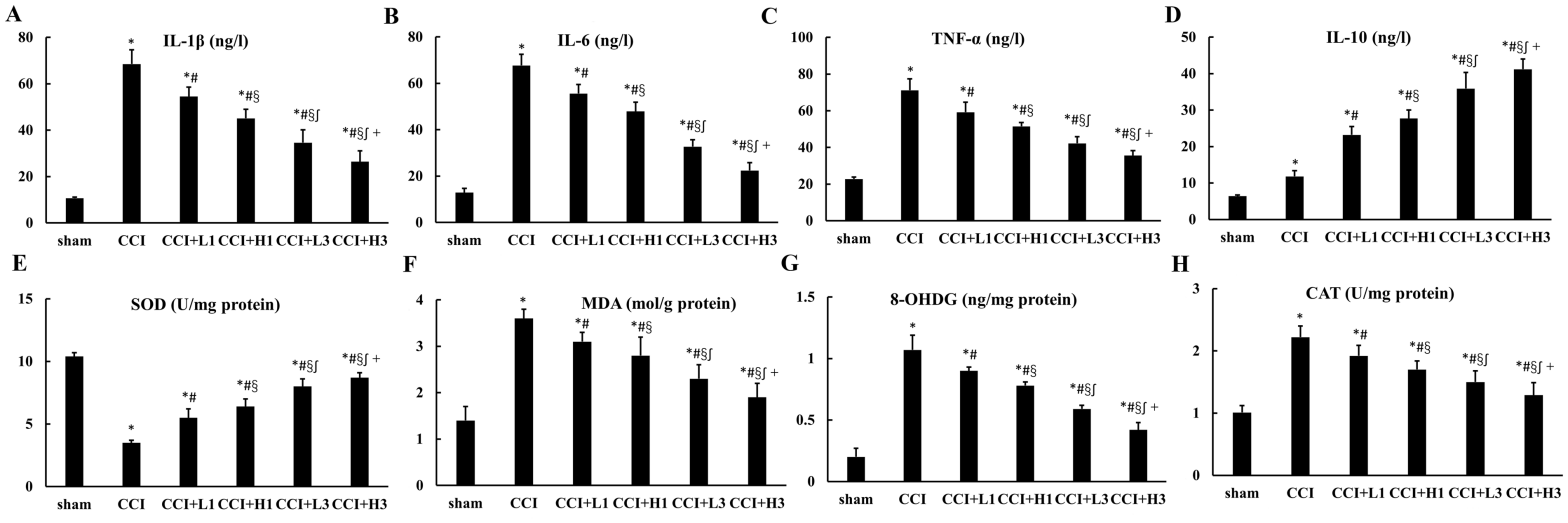
The inflammation and oxidative stress injury in DRG (*n* = 6, means ± SD). **(A)** IL-1β; **(B)** IL-6; **(C)** TNF-α; **(D)** IL-10; **(E)** SOD; **(F)** MDA; **(G)** 8-OHDG; **(H)** CAT. CCI: chronic constriction injury; IL: interlenkin; TNF: tumor necrosis factor; SOD: superoxide dismutase; MDA: malondialdehyde; 8-OHDG: 8-hydroxydeoxyguanosine; CAT: catalase. ^*^p < 0.05 vs. sham group; ^#^*p* < 0.05 vs. CCI group; ^§^*p* < 0.05 vs. CCI + L1 group; ^∫^*p* < 0.05 vs. CCI + H1 group; ^+^*p* < 0.05 vs. CCI + L3 group.

Differences between multiple groups were analyzed by one-way ANOVA and Bonferroni test was used for post comparisons between groups. Compared with the sham group, the levels of IL-1β, IL-6, and TNF-*α* in the sciatic nerve were increased significantly in the CCI, CCI + L1, CCI + H1, CCI + L3, and CCI + H3 groups (*p* < 0.05). Compared with those in the CCI group, the levels of IL-1β, IL-6, and TNF-α in nerve were decreased in the CCI + L1, CCI + H1, CCI + L3, and CCI + H3 groups (*p* < 0.05). Additionally, the levels of IL-1β, IL-6, and TNF-α in the sciatic nerve were lower in the CCI + L3 and CCI + H3 groups than in the CCI + L1 and CCI + H1 groups, and those in the CCI + H3 group were lower than those in the CCI + L3group (*p* < 0.05). Furthermore, the level of IL-10 in the sciatic nerve showed an opposite trend to that of IL-1β, IL-6, and TNF-α (*p* < 0.05; Figure 4). In addition, the levels of IL-1β, IL-6, TNF-α, and IL-10 in the DRG exhibited results similar to those in the sciatic nerve (Figure 5).

### 3.5 NHW decreased CCI-induced oxidative stress injury

Differences between multiple groups were analyzed by one-way ANOVA and Bonferroni test was used for post comparisons between groups. Compared with those in the sham group, the levels of MDA and 8-OHDG in the sciatic nerve were significantly greater in the CCI, CCI + L1, CCI + H1, CCI + L3, and CCI + H3 groups (*p* < 0.05). Compared with those in the CCI group, the levels of MDA and 8-OHDG in the sciatic nerve were decreased in the CCI + L1, CCI + H1, CCI + L3, and CCI + H3 groups (*p* < 0.05). Additionally, the levels of MDA and 8-OHDG in the sciatic nerve were lower in the CCI + L3 and CCI + H3 groups than in the CCI + L1 and CCI + H1 groups, and those in the CCI + H3 group were lower than those in the CCI + L3 group were (*p* < 0.05). Furthermore, the SOD and CAT levels in the sciatic nerve tended to be opposite those of MDA and 8-OHDG (*p* < 0.05; Figure 4). In addition, the levels of SOD, CAT, MDA and 8-OHDG in the DRG were similar to those in the sciatic nerve (Figure 5).

### 3.6 NHW activated the Nrf2/HO-1-sulfiredoxin1 pathway

**Figure 6:**
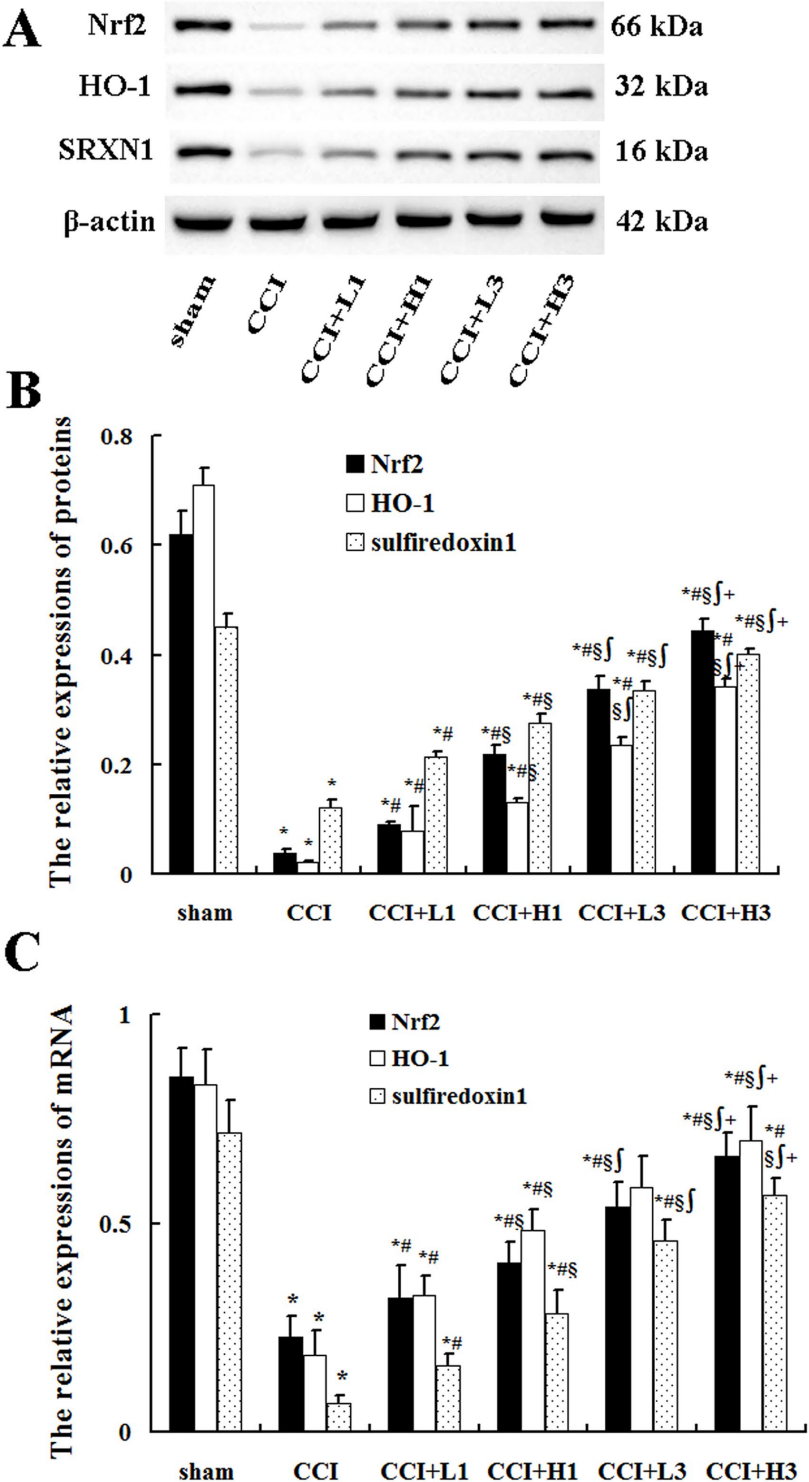
Nrf2, HO-1, and sulfiredoxin1 protein and mRNA expression in the DRG (*n* = 4, mean ± SD). **(A)** Nrf2, HO-1, and sulfiredoxin1 proteins; **(B)** relative expression levels of Nrf2, HO-1, and sulfiredoxin1 proteins; **(C)** relative expressions of Nrf2 mRNA, HO-1 mRNA, and sulfiredoxin1 mRNA. SRXN1: sulfiredoxin1. ^*^p < 0.05 vs. the sham group; ^#^*p* < 0.05 vs. the CCI group; ^§^*p* < 0.05 vs. the CCI + L1 group; ^∫^*p* < 0.05 vs. the CCI + H1 group; ^+^*p* < 0.05 vs. the CCI + H1 group.

Differences between multiple groups were analyzed by one-way ANOVA and Bonferroni test was used for post comparisons between groups. Compared with those in the sham group, the expression of Nrf2, HO-1 and sulfiredoxin1 proteins in the DRG was significantly lower in the CCI, CCI + L1, CCI + H1, CCI + L3, and CCI + H3 groups (*p* < 0.05). Compared with those in the CCI group, the Nrf2, HO-1 and sulfiredoxin1 protein levels in the DRG were increased in the CCI + L1, CCI + H1, CCI + L3, and CCI + H3 groups (*p* < 0.05). Additionally, compared with those in the CCI + L1 and CCI + H1 groups, the expression of Nrf2, HO-1 and sulfiredoxin1 proteins in the CCI + L3 and CCI + H3 groups increased, and the expression in the CCI + H3 group was greater than that in the CCI + L3 group (*p* < 0.05; Figure 6). In addition, the expression levels of Nrf2 mRNA, HO-1 mRNA and sulfiredoxin1 mRNA in the DRG were similar results to those of the relative proteins (Figure 6).

### 3.7 Additional experiment

**Figure 7:**
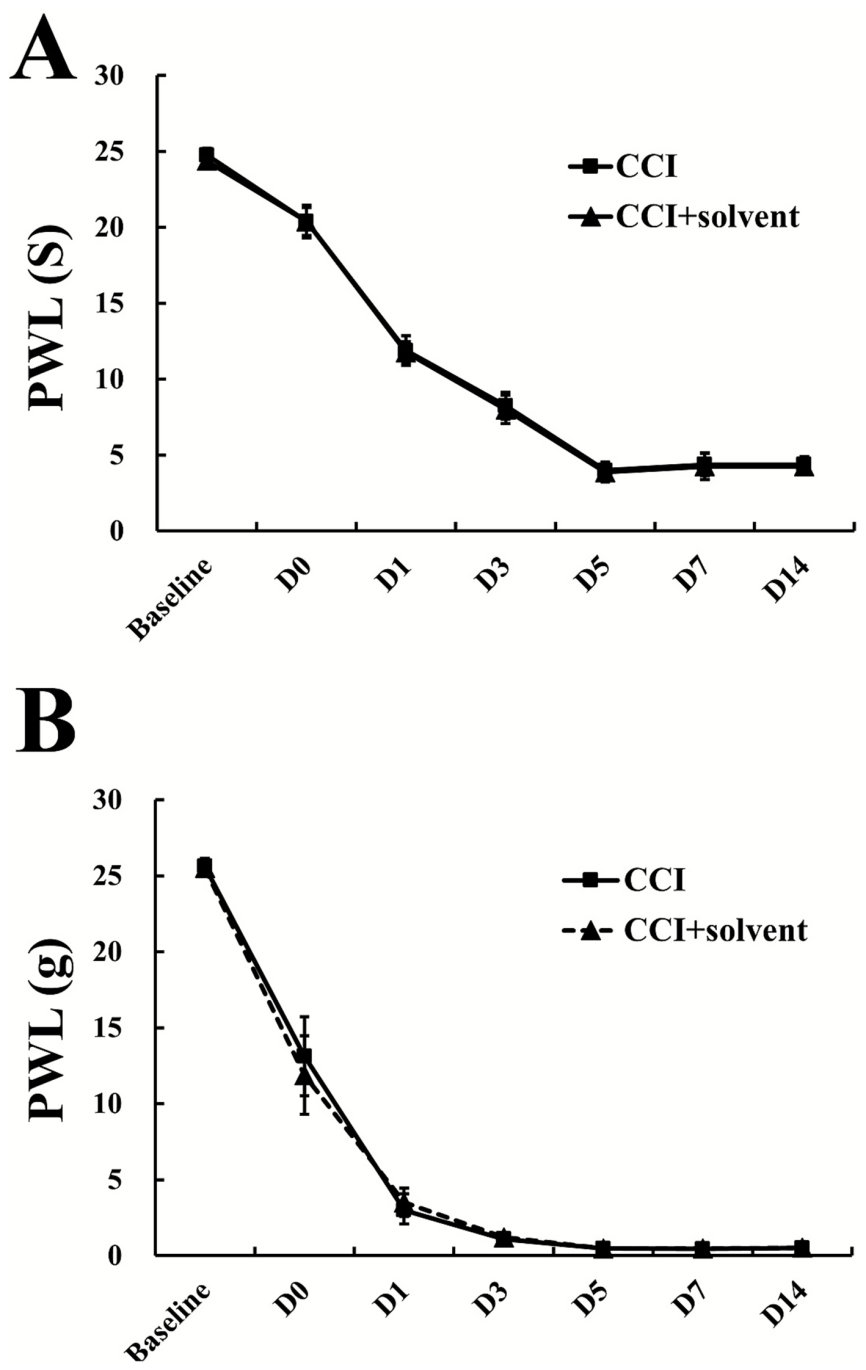
Additional experiments for behavioral test of CCI and CCI + solvent (*n* = 6; means ± SD). **(A)** Paw withdrawal threshold (PWT); **(B)** paw withdrawal latency (PWL).

In order to exclude the influence of solvent injection, we conducted additional experiments and confirmed that solvent injection did not affect the neuropathic pain caused by CCI. At baseline, and Day 0, 1, 3, 5, 7, and 14 after CCI, the PWT and PWL in CCI group and CCI + solvent group were similar without significant difference (Figure 7).

## 4 Discussion

In this study, the NHW contained H_2_ bubbles less than 1 μm in diameter and a lysis state of H_2_ and was used in the rat CCI model. The results showed that NHW attenuated the sciatic nerve injury, reduced PWT and PWL, decreased inflammation and oxidative stress injury to the nerve and DRG, and activated the Nrf2/HO-1-sulfiredoxin1 pathway.

### 4.1 NHW attenuated behavioral tests in CCI rats

NHW alleviated CCI-induced mechanical allodynia and thermal hyperalgesia, as determined by PWT and PWL. Administering a low or high concentration of NHW once after CCI can decrease PWT and PWL, providing protection against mechanical allodynia and thermal hyperalgesia. These findings indicated that early administration of NHW not only alleviated neuropathic pain but also inhibited the development of NP. Of course, repeated administration of high concentrations of NHW improved the protective effects against mechanical allodynia and thermal hyperalgesia.

### 4.2 NHW attenuated inflammation and oxidative stress injury in CCI rats

IL-1β is a potent pro-inflammatory cytokine expressed by monocytes, macrophages, and dendritic cells and plays a critical role in inflammation and immune responses (Arbelaez-Bonozo et al., 2024). IL-6 is a cytokine in the chemokine family (Lee et al., 2023). TNF-*α* can regulate the function of some immune cells and participate in the pathogenesis of diseases (Tian et al., 2024). In addition, TNF-α transmits information to the nucleus through specific receptors on the cell membrane, promoting inflammation (Tian et al., 2024). IL-1β can induce the release of IL-6 and TNF-α and stimulate T-cell activation, which can stimulate local and systemic inflammatory responses (Li et al., 2022). In this study, local injection of NHW decreased the levels of IL-1β, IL-6, and TNF-α in the nerve and DRG and increased the level of the anti-inflammatory factor IL-10 to inhibit the inflammatory response. This finding indicated that local injection of NHW not only decreased nerve inflammation but also downregulated inflammation in the DRG.

Oxidative stress refers to the process in which, under certain special conditions, a disruption of the redox balance in the body results in the production of free radicals that exceed the body’s antioxidant clearance capacity, leading to the accumulation of reactive oxygen species in cells and organs, thereby causing oxidative stress injury (Zhang et al., 2024). MDA is the main product of lipid peroxidation damage (Ambe et al., 2024). 8-OHDG is a biomarker of endogenous and exogenous factors affecting DNA oxidative damage (Lazutka et al., 2024). The detection of 8-OHDG can be used to evaluate the degree of oxidative damage and repair *in vivo*, as well as the relationship between oxidative stress and DNA damage (Lazutka et al., 2024). SOD primarily functions to catalyze the dismutation of superoxide anion radicals into hydrogen peroxide and oxygen (Sahranavard et al., 2025). It is the most important and optimal free radical scavenger in the body and maintains metabolic balance (Sahranavard et al., 2025). CAT is also an important antioxidant enzyme that catalyzes the decomposition of hydrogen peroxide to produce water and oxygen, thereby protecting cells from oxidative stress damage (Zhang et al., 2023). The inhibitory effect of many drugs on oxidative stress is achieved through the enhancement of SOD and CAT activity. In this study, local injection of NHW inhibited oxidative stress injury by elevating SOD and CAT activity and reducing MDA and 8-OHDG levels in the sciatic nerve and the DRG. This finding indicated that local injection of NHW not only decreased nerve oxidative stress injury, but also inhibited oxidative stress injury in the DRG.

In 2013, Chen et al. reported that H_2_-rich water given by intrathecal injection can decrease oxidative stress injury to alleviate NP in a rat CCI model (Chen et al., 2013). In 2014, Kawaguchi et al. reported that drinking H_2_ water can alleviate both allodynia and hyperalgesia in a mouse CCI model by suppressing oxidative stress in the DRG (Kawaguchi et al., 2014). In 2018, Wang et al. reported that intraperitoneal injection of H_2_-rich water also improved PWT and PWL in a rat CCI model (Wang et al., 2018). In 2022, Martínez-Serrat et al. demonstrated that intraperitoneal injection of H_2_-rich water inhibited inflammatory reactions and oxidative stress injury in the DRG in a mouse CCI model (Martínez-Serrat et al., 2022). These results consistently demonstrate that H_2_ can play a role in reducing inflammatory reactions and oxidative stress injury, and different administration methods can affect these processes. This study showed similar results, and this study was the first to demonstrate that ultrasound-guided local injection of NHW can alleviate CCI-induced NP in rats.

### 4.3 Advantages of NHW

Compared with intrathecal injection, drinking, and intraperitoneal injection, local injection of NHW is more effective, and this approach is closer to standard clinical applications. Furthermore, compared with continuous or multiple applications in previous studies (Martínez-Serrat et al., 2022; Wang et al., 2018; Kawaguchi et al., 2014), in this study, we showed that three applications of NHW also had a good protective effect against CCI-induced NP, which means that the effect of NHW has a certain long-term stability. This study also revealed that local injection of NHW not only reduced nerve damage but also affected DRG protein expression and alleviated pain.

In this study, we also observed the effects of the concentration and use frequency of NHW on CCI. First, the protective effects of high concentrations of NHW on inflammation, oxidative stress injury, nerve injury, mechanical allodynia, and thermal hyperalgesia were greater than those of low concentrations. Second, whether at low or high concentrations, early intermittent use of NHW 3 times had a stronger protective effect than using it only once. These findings indicated that the protective effects against CCI-induced injury may be dose-dependent and that repeated administration of NHW resulted in better outcomes; in addition, no adverse reactions were observed.

### 4.4 The Nrf2/HO-1 pathway involved in the NHW effects

Nrf2 is the main regulatory factor of the antioxidant defense system in the body and is involved in signal transduction related to various intracellular defense mechanisms (Zeng et al., 2024). HO-1 is a downstream target protein of Nrf2 and degrade hemoglobin and release biliverdin, CO, and ferrous ions (Hua et al., 2024). HO-1 and its products play beneficial roles by protecting against oxidative damage and modulating inflammatory responses. When tissues/cells are stimulated by injury or are in a stress state, HO-1 can respond through Nrf2 regulation, with highly upregulated expression levels and significant antioxidant effects. The complete Nrf2/HO-1 pathway exerts various effects, such as antioxidant and anti-inflammatory effects, maintenance of mitochondrial homeostasis, and inhibition of apoptosis, ultimately affecting diseases outcomes (Nan et al., 2024). Sulfiredoxin1, an endogenous antioxidant protein, plays an important role in cells and is involved mainly in protein repair and the oxidative stress response. It can resist oxidative stress damage (Wang et al., 2022).

Firdoos et al. reported that upregulating Nrf2 and HO-1 expression relieved rat CCI-induced mechanical allodynia and thermal hyperalgesia, decreased inflammatory cascades, and elevated antioxidant enzyme levels (Firdoos et al., 2024). Zhu et al. also demonstrated that CCI-induced mechanical allodynia, thermal hyperalgesia, neuroinflammation, and oxidative stress were associated with the inhibition of the Nrf2/HO-1 signaling pathway (Zhu et al., 2023). In 2022, our previous study proposed that the Nrf2/HO-1 pathway may exert antioxidant and anti-inflammatory effects through sulfiredoxin1 in a rat lung ischemia–reperfusion injury model (Wang et al., 2022). In this study, NHW upregulated the protein expression of Nrf2/HO-1 and sulfiredoxin1 and attenuated CCI-induced NP, indicating that the protective effects of NHW on rat CCI-induced NP may be related to the Nrf2/HO-1-sulfiredoxin1 pathway.

## 5 Limitations

This study has several limitations. First, this study revealed that in addition to affecting nerves, NHW can also regulate the expression of DRG proteins, but we have not explored how NHW acts on the DRG. We speculate that this phenomenon may be related to the high tissue permeability of H_2_. Second, whether NHW selectively acts on DRGs associated with damaged nerves has not been explored, and the L4 DRGs and L6 DRGs, and the changes in protein expression in the central spinal cord have not been explored. In addition, we did not confirm the concentration of H_2_ in all the NHW samples. However, we used the equipment to repeatedly produce about six samples of NHW, and the concentration can reach 2,600 ppb. This sample was used in the high-concentration group, and the same sample was diluted 1 time (1,300 ppb) for use in the low-concentration group. Therefore, although a concentration of 2,600 or 1,300 ppb cannot be guaranteed completely, the concentration in the low concentration group can be guaranteed to be lower than in the high concentration group. Finally, this study was conducted for only 14 days, and no inhibitors were used to clarify the Nrf2/HO-1-sulfiredoxin1 pathway; and this effect and mechanism of action of NHW were not confirmed in cells and will be explored in future study.

## 6 Conclusion

Ultrasound-guided early local injection of NHW attenuated sciatic nerve injury, alleviated mechanical allodynia and thermal hyperalgesia, and inhibited inflammation and oxidative stress damage in the rat model of CCI, which involving Nrf2/HO-1-sulfiredoxin1 pathway.

## Data Availability

The original contributions presented in the study are included in the article/supplementary material, further inquiries can be directed to the corresponding author.
